# Shorter Lines Facilitate Reading in Those Who Struggle

**DOI:** 10.1371/journal.pone.0071161

**Published:** 2013-08-05

**Authors:** Matthew H. Schneps, Jenny M. Thomson, Gerhard Sonnert, Marc Pomplun, Chen Chen, Amanda Heffner-Wong

**Affiliations:** 1 Science Education Department, Harvard-Smithsonian Center for Astrophysics, Cambridge, Massachusetts, United States of America; 2 Harvard Graduate School of Education, Cambridge, Massachusetts, United States of America; 3 Department of Computer Science, University of Massachusetts at Boston, Boston, Massachusetts, United States of America; Harvard Medical School, United States of America

## Abstract

People with dyslexia, who ordinarily struggle to read, sometimes remark that reading is easier when e-readers are used. Here, we used eye tracking to observe high school students with dyslexia as they read using these devices. Among the factors investigated, we found that reading using a small device resulted in substantial benefits, improving reading speeds by 27%, reducing the number of fixations by 11%, and importantly, reducing the number of regressive saccades by more than a factor of 2, with no cost to comprehension. Given that an expected trade-off between horizontal and vertical regression was not observed when line lengths were altered, we speculate that these effects occur because sluggish attention spreads perception to the left as the gaze shifts during reading. Short lines eliminate crowded text to the left, reducing regression. The effects of attention modulation by the hand, and of increased letter spacing to reduce crowding, were also found to modulate the oculomotor dynamics in reading, but whether these factors resulted in benefits or costs depended on characteristics, such as visual attention span, that varied within our sample.

## Introduction

The widespread adoption of e-readers is driving a fast-moving evolution in the social conventions for reading, opening the door to the possibility that new methods for reading may begin to take hold. Given that an estimated 5% to 17% of all people struggle with the system of reading currently in place [1], it is reasonable to ask whether this evolution in reading can lead to new methods that are better matched to the atypical neurology of those with dyslexia and thus might ameliorate its detrimental effects.

Anecdotally, people with dyslexia sometimes remark that reading on e-readers seems easier, and researchers have long suspected that altered treatments of text may produce beneficial effects in those who struggle to read. Among measures proposed in the past are alterations to fonts [2], [3], rearrangements in the physical formatting of the text [4], and masking to isolate attention [5]. While, in some cases, benefits have been observed, these effects have generally been small and, occasionally, controversial and difficult to reproduce.

This state of research encourages the investigations undertaken here to ascertain whether alternate methods for reading, made practical by use of e-readers, can address known impairments in dyslexia, such as poor oculomotor control [6], and deficits in visuospatial attention [7]. In this paper, we use eye tracking to observe high school students with dyslexia as they read using popular handheld portable reading devices. Our experiment varies a number of factors that differentiate such devices from traditional presentations of text. It investigates the effects of the device being held in the hand, of differing linewidths (which result from devices of different sizes), and of the alteration of inter-letter spacing.

**Figure 1:**
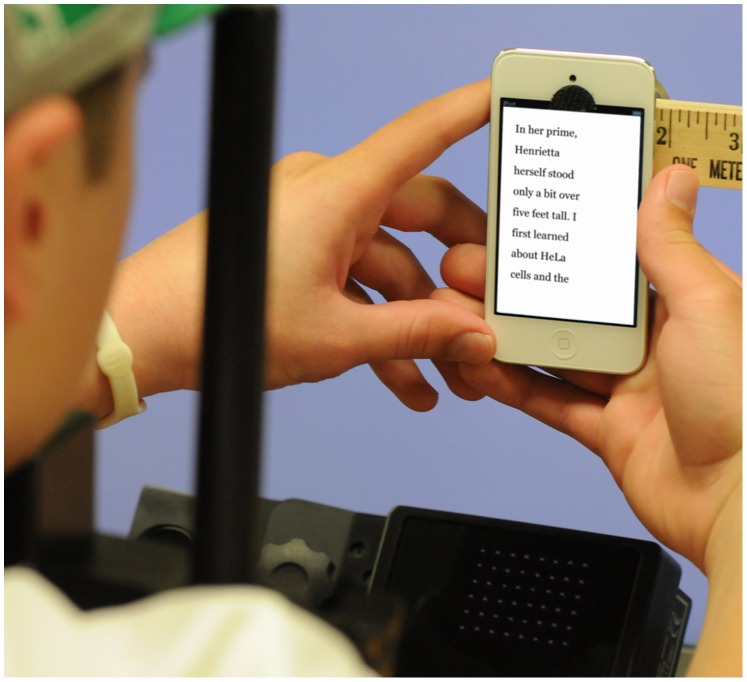
Reading on the Apple iPod Touch using the hands. The device is suspended from a stable mount in front of the participant, while eye movements are observed using an eye tracking device (partially visible toward the bottom of the photo). In this condition, the participant holds the device. In the NO-HAND condition, the participant’s hands are placed in his or her lap. The PAD condition is similar, except that the iPod is replaced with the larger format Apple iPad. (Photo: Randy H. Goodman).

Effects of the hand are interesting because mobile reading devices are typically used while held in the hand, and attentional processes are biased by proximity to the hand [8], shielding visual perception from interference by attention [9] and enhancing sensitivity to detail [10]. Dyslexia is associated with numerous deficits in visual attention [7], [11]–[13], and therefore it is an interesting question whether holding text in the hand influences reading in people with dyslexia. Our experiment therefore varies whether reading is performed while the reading device is held near the hands, or not (see Fig. 1).

**Figure 2:**
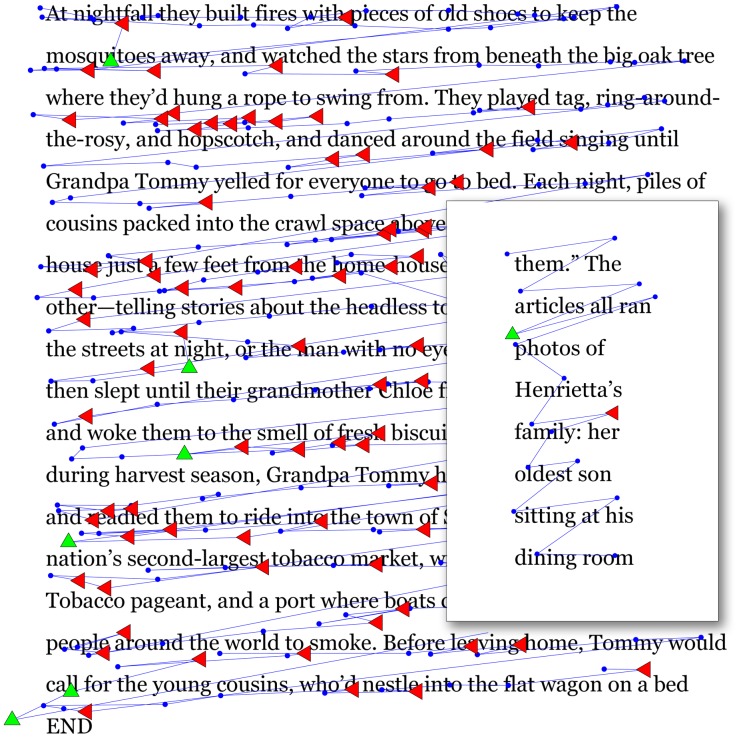
PAD and POD conditions compared. Participants read 208 words per trial, two trials per condition, in each of eight unique combinations of conditions. In a single trial all 208 words are displayed on a single page in the PAD condition, while in the POD condition (inset overlaid) 12 pages are required to display the same number of words. The figure superimposes gaze-tracking data sampled from the *same participant*. Blue dots indicate fixations. Leftward regressions (LEFT) are marked in red, while symbols in green are regressions directed upwards (UP). Note that the density of horizontal regressions is higher in the PAD condition compared with POD. This individual makes numerous leftward regressions –almost as if automatic– in the PAD condition. However, in the POD condition, the density of leftward regressions is reduced. Importantly, the figure illustrates that this reduction in horizontal regressions does not occur at the expense of regressions upwards.

Another question we investigate pertains to the small physical size of many mobile reading devices (e.g., smart phones). It has been suggested that the small reading window used in such devices may facilitate reading in dyslexia by limiting the span of attention required for reading [4]. To investigate this, participants with dyslexia read using either an Apple iPad (PAD condition) or a smaller Apple iPod Touch (POD condition), keeping the angular dimensions of the characters and the spacing of the lines identical in both conditions (see Fig. 2).

**Figure 3:**
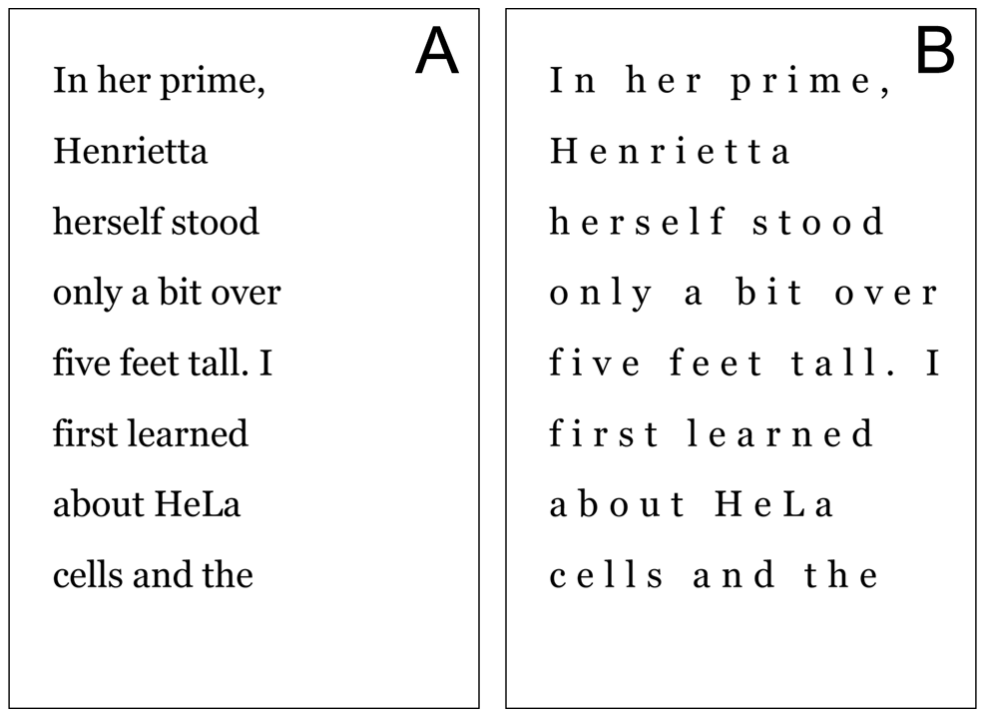
NORMAL and SPACED conditions compared. Extra wide letter spacing is used in the SPACED condition (B). Linewidth is here defined as the number of words per line, and this is held constant in each condition. Line spacing is also held fixed.

Finally, a third variable investigates the effects of letter spacing. An earlier study found that increasing inter-letter spacing facilitates reading in children with dyslexia [14]. Increased letter spacing reduces neurological interactions that occur between letters to impede recognition, an effect known as crowding, which has been observed to be heightened in dyslexia [15], [16]. Here, NORMAL letter-spacing is compared with SPACED, wherein letter-spacing is increased (see Fig. 3). To account for an important confound of line spacing in the experiments of [14], in our experiments line spacing is held constant.

## Methods

### Ethics Statement

This study was approved by the Committee on the Use of Human Subjects in Research at Harvard University. In accordance with the approved research protocol, volunteers who were not minors provided written informed consent, while those who were minors provided written assent, with additional written consent obtained from their parents or guardians.

### Research Design

The goal of our study was to investigate whether and how differences in e-reader technologies and set-up affect reading in dyslexia. We used a within-subjects design to focus analysis on effects resulting from manipulations of the device, an accepted technique used in this field (e.g., [17]). The reading device (PAD/POD), inter-letter spacing (NORMAL/SPACED), and involvement of the hands (HAND/NO-HAND) are the conditions varied experimentally in a balanced (2×2×2) design of the “repeated measures” type, in which all participants were measured in all conditions, so that participants served as their own controls.

### Participants

Participants were 27 (13 M/14 F) students with lifelong histories of reading struggles. These were high school students enrolled at Landmark High School, in Prides Crossing, MA (USA), a school exclusively for students with language disabilities, except for one participant (8th grade) who was from another school. All students had vision that was normal, or corrected to normal, and no histories of neurological disorders. The current literacy and phonological awareness profile of each participants was measured prior to the experiment using the Test of Word Reading Efficiency (TOWRE; [18]), the Gates-MacGinitie Reading Tests [19] and three subtests of the Comprehensive Test of Phonological Processing (CTOPP; [20]). Participants’ non-verbal ability was measured using the Block Design subtest of the Wechsler Abbreviated Scale of Intelligence; WASI [21]. The observed characteristics of the group are summarized in Table 1.

**Table 1:** Descriptive statistics for participants’ demographic and measurement information.

Variable	Obs	Mean	Std	Min	Max
Grade	25	10.56	0.96	9	12
Gender (0 = male; 1 = female)	27	0.52	0.51	0	1
Age	25	17.12	1.05	15	19
***Standardized Scores***					
Block Design	22	49.91	9.42	28	62
Elision	26	9.15	1.93	4	12
Memory for Digits	26	8.31	2.55	2	14
Rapid Letter Naming	26	6.37	2.13	2	10
Rapid Digit Naming	26	7.48	2.52	2	13
Sight Word Efficiency	26	78.04	11.16	54	100
Phoneme Decoding Efficiency	26	79.31	9.05	60	96
Degrees of Reading Power Level*	25	59.2	12.69	34	77
Gates-MacGinitieReading Test**	27	546.33	22.49	500	592

* DRP supplied by school.

** G-M Level 10 reported.

### Stimuli

Participants read material excerpted from an age-appropriate non-fiction book on biomedical science [22]. Passages consisted of 208 words and were randomly selected from the book, avoiding segments containing unusual formatting or extensive dialogue. Each passage began at the start of a sentence, and ended after 208 words, appending the word “END.” Sixteen such passages were selected, in addition to one reserved for practice. All text was formatted using a 32-pt Georgia font, with a line spacing that was increased by a factor of 1.7 compared with normal spacing. Margins were left justified and right ragged, and the width of the margin was chosen to avoid the possibility that words would become hyphenated. Two versions of the text (NORMAL/SPACED) were prepared for each device (PAD/POD). Text for the POD condition was packed to accommodate the screen dimensions of the Apple iPod Touch in portrait mode (5 cm×7.5 cm), while text for the PAD condition was dimensioned to fit the Apple iPad in landscape mode (19.7 cm×14.8 cm), keeping the angular dimensions of the characters and their spacing identical in both conditions (see Fig. 2). Using this formatting, a 208-word passage could be displayed on a single PAD page. However, owing to the smaller size of the iPod screen, in order to keep the angular dimensions of the characters identical in the PAD and POD conditions, 12 POD pages were required to display the same content as one PAD page. Effects of crowding were varied (NORMAL/SPACED) by increasing character spacing by 29% in the SPACED condition. Line breaks were manually inserted to ensure that the number of words presented in each line (here defined as “linewidth”) remained identical in the two spacing conditions (see Fig. 3).

### Apparatus

Reading material prepared for the POD condition was preloaded on an unmodified third generation Apple iPod Touch. The device had a screen resolution of 640×960 pixels at 128 pixels per cm. Passages for the PAD condition were presented on an Apple iPad 2, at a resolution of 1024×768 pixels, at 52 pixels per cm. In both cases, the Apple iBook app was used to display the text, stored on the device in pdf format, to preserve font and spacing. The screens were adjusted for a black level of 0.9 cd/m^2^ and a white level of 66 cd/m^2^ for the POD condition, and 0.1 cd/m^2^ and 70 cd/m^2^, respectively, for the PAD condition. Ambient room luminosity was adjusted between 1 Lux and 280 Lux, depending on the needs of the participant, to maintain a pupil size optimal for eye tracking.

The iPad or iPod was suspended in front of a 23-inch Apple Cinema (493 mm×308 mm) flat-screen LCD monitor that served as a calibration screen. This was placed at a distance of 60 cm from the participants, set to a white level luminance of 66 cd/m^2^. The iPad or iPod was mounted in front of this calibration screen at a distance of 35 cm from the participant, positioned so as not to obscure the eye tracking camera or its illuminator (see Fig. 1). The device was held by an adjustable mount that facilitated removal for calibration and for swapping of devices to between the PAD and POD conditions.

Eye movement data were acquired and recorded via a desk mounted Eye-Link 1000 system (SR-Research, Ontario, Canada) running at a sampling rate of 1000 Hz. Calibration and data acquisition were controlled by custom software. Because the iPad and iPod could only be controlled via their touchscreens, the displays were manually controlled by a technician who interacted with the device using a stylus. During data acquisition, a page-turn target (black square) was continuously visible on the calibration screen behind the iPad or iPod, positioned to appear just above the top edge of the reading device. When participants directed their gaze to this target, this action was interpreted in real time by the data acquisition software to trigger an audible signal that directed the technician to quickly update the iPad or iPod display.

During reading, participants sat in a chair, and a chin rest was used to stabilize the head. The position of the participants’ hands was controlled as a condition of the experiment (HAND/NO-HAND). In the NO-HAND condition participants held a tennis ball with both hands placed in their laps. In the HAND condition participants placed each hand on either side of the iPod/iPad device (see Fig. 1). Elbow supports helped prevent blockage of the eye-tracking camera located beneath the reading device.

### Procedures

Device (PAD/POD), crowding (NORMAL/SPACED), and hand-position (HAND/NO-HAND) were varied in a (2×2×2) design that yielded eight possible unique combinations of conditions. Each of the eight conditions involved a practice followed by two reading trials, each presenting a different 208-word passage. To control for bias due to order, these combinations were distributed among participants using a pair of 8×8 mutually orthogonal Latin squares [23], one for passages 1–8, the other for passages 9–16. This arrangement was repeated for each group of eight participants. In total, all participants read all of the same 16 reading passages, but the order of these passages and their assignments to conditions varied according to the mutually orthogonal Latin square design.

Prior to reading, whenever a device configuration was changed, a standard (typically, nine-point) calibration was performed. Reading trials commenced when the participant issued a signal to the technician (by directing their gaze to the page-turn target) to advance the page on the iPod or iPad. When participants reached the end of the displayed text, they again directed their gaze to the page-turn target, and the technician advanced the page. In the POD condition, reading the entire 208-word passage required 12 such page turns, while the PAD condition required only one. Participants read all text silently. When reading of the entire 208-word passage was completed, participants were asked to recall the content of the passage read, and their description was rated for fidelity (FIDELITY), coding for the number of substantive details recalled using a four-point scale (0–3). A score of zero indicated the participant was unable to recall any details, while a score of three indicated three or more details were described.

### Eye Motion Analysis

Standard software (EDF Converter; SR-Research, Ontario, CA) was used to extract fixation parameters from the eye data. Custom software written in Matlab (Mathworks, Natick, MA) was used to fit the fixation positions to the distribution of words in the text for each trial. Here, an objective algorithm scaled and rotated the XY coordinates of eye fixation data to match the overall pattern of words displayed in each trial. Upward directed eye movements off the screen, toward the page-turn target, were used to estimate the approximate times reading began and ended in each trial. The algorithm then used this information to disambiguate data corresponding to each of the 12 pages in the POD condition, and the single page in the PAD condition. Once this was known, the algorithm refined initial estimates for the start and end of reading by identifying the time-stamps of gaze fixations landing closest to the positions of the first and last words on the page.

Automated fit to the data was examined for each page. Cases where the automatic algorithm failed to converge, or where the eye tracker failed to hold lock on the gaze (for example when participants inadvertently blocked the camera during reading), were flagged and omitted from subsequent analysis. In some instances, when it was clear that the fit failed for reasons easily evident and corrected (for example, if the end of reading was lost because participants forgot to signal a page turn, or if erratic eye movements at the start of a page confused the algorithm), it was possible to correct such errors by inspection and restore the convergence of fit.

Given that it takes time for a subject to locate the first word on a page and settle into a stable pattern of reading, the timestamp of the initial fixation corresponding to the first word in the text (whether or not corrected by inspection) was not assumed to be a reliable measure for estimating speed of reading. Similarly, when participants approached the end of a page, procedures required them to engage in special actions (e.g., to gaze at the page-turn target) that could potentially disrupt their reading. For this reason, the automated estimates of when the last word is read were also considered unreliable. To counteract such effects, we based the measurements we report on parameters observed over a “trimmed” reading interval that ignores a fixed span of text at the start and end of each page. To do this, the objective algorithm matched gaze positions to the third word from the start and the third word from the end of each POD page, and the 25th word from the start and 25th word from the end for each PAD page, so that reading measures were based on reading a total of 160 words for each passage, rather than 208.

**Figure 4:**
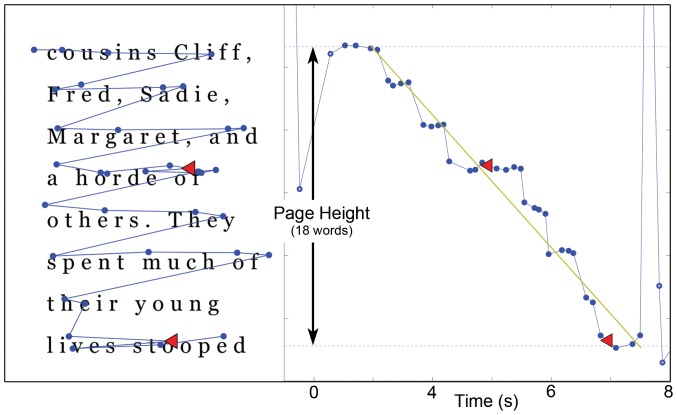
Sample eye tracking data for POD SPACED HAND condition. Fixation positions are fit to text displayed on the iPod Touch (left panel). The experiment counts the number of fixations (blue dots), regressions (red), and reading speed. Speed is determined by examining the vertical component of the gaze, plotted as a function of time in the left panel. Data in an interval near the start and end of each page is ignored to avoid boundary effects between pages, and a robust linear fit to this data (green) used to compute an instantaneous measure of reading rate.

We observed eye motion statistics over the trimmed time interval to compute fixation counts, fixation durations, and errors in oculomotor efficiency (e.g., number of regressive saccades) and distractibility (i.e., glances off-page). For reading speed, we introduced a novel reading rate measure that performs a robust linear regression on the y-coordinate of the gaze as a function of time (see Fig. 4). This measure is robust against outliers in motion, and results in a measure of reading rates that is likely more stable and reliable than that computed from traditional time interval measures. (See detailed description of variables below.)

### Visual Attention Span (VAS)

Response to a global letter report task (sometimes referred to as the “visual attention span”) was measured using custom software (iCue) on the iPod device, adapting procedures described in [24]. The participant held the iPod in the hand, at a comfortable reading distance. The participant manually started each trial by tapping the device touchscreen. This initiated a 1000 ms presentation of a number (1–10) centrally-placed on the screen that the participant read aloud (used to facilitate score-keeping). Following this, a blank screen appeared for 1000 ms, and then a centrally placed fixation marker, held for 1000 ms. Fixation was followed by a second blank screen of 500 ms duration. A six-letter global report task followed immediately, wherein 6 unique characters, each separated by four spaces, were chosen with no order constraint from among (B, P, T, F, L, M, D, S, R, H), and displayed on screen for 200 ms using a 20 pt fixed-width Courier font. The string of letters spanned 4 cm on the display. The letter string presented in each trial was unique. Following the global report stimulus, a blank screen appeared, at which point participants reported any letters recalled, irrespective of order, with no constraint on time. Following a practice session, 24 trials were presented. The number of correctly identified letters was totaled for each trial, and a mean was computed to create a score for each participant (VAS).

### Variables

This study tested the hypotheses that reading is facilitated when (a) text is formatted in a narrow window (POD condition), (b) letter-spacing is increased to reduce crowding (SPACED condition), and (c) text is placed in proximity to the hand (HAND condition). Improved reading efficiency is taken to be indicated by (i) higher reading speed, (ii) fewer fixations, (iii) fewer ineffective eye movements (e.g., regressive saccades), or (iv) better reading comprehension.

The measured slope of the regression line indicating the average vertical angular velocity of the gaze in pixels per sec was re-normalized to obtain a reading rate: It was multiplied by the number of words covered in the vertical gaze span to construct a novel robust measure of reading rate (RATE: words/min). Fixation parameters observed include the number of fixations recorded during the trimmed interval (FIX) and counts of the number of irregular eye movements made during reading. These included regressive saccades, indicating that the participant looked back while reading (LEFT), eye movements made to lines above (UP) or below (DN) the location of the expected next word, and eye movements directed off the page (OUT), indicative of distraction. These tracking error variables were summed to compute an overall measure of tracking efficiency (TOT).

Two variables measured reading comprehension: post-test assessments of reading fidelity as a dependent variable, and an a-priori indicator of general reading power as a predictor variable. Given that the sample of participants was drawn from a special school that provides a strong focus on reading intervention, and that many of these high school students were enrolled in this school for a significant portion of their academic careers, it is not surprising that more than 90% of the Fidelity responses were scored at 3, indicating that students recalled the content with a high degree of fidelity. (In fact, students typically described so many details during interview, extra time spent on student descriptions unexpectedly became a driving factor in scheduling participants.) Because a ceiling effect skewed the response distribution and created a highly non-normal shape, with the bulk of the observations being at the extreme, we separated the instances where comprehension essentially failed from those where students read well, by recoding this variable as a low score (0–1), and a high score (2–3), to produce the dichotomous reading comprehension measure used in our analysis (FIDELITY: low/high). In an effort to control for the participants’ general level of reading comprehension, we used a-priori reading measures supplied by the school (Degrees of Reading Power from the Connecticut Mastery Test [25]) to construct a predictor variable from a median split in these scores (DRP: high/low). Students with a DRP score of 61 or higher were assigned to the “high reading” group; students with a DRP score below 61, the “low reading” group.

For technical reasons, not all eye-tracking trials resulted in valid measurements. In some cases, especially in the HAND condition, the participant’s arm would block the eye tracking camera, causing dropouts in recordings, while in other instances pressure from the hand dislodged the reading device. Other issues that influenced the quality of the recording included cases where students exhibited patterns of reading that were so erratic that the automated algorithm used to match the text to the fixation positions failed to reliably converge. The experimenters therefore inspected each of the trials and rated its validity as good, acceptable, or poor. The trials of the last category were omitted from analysis. This meant a decrease in usable trials from 2654 to 2178 (a 17.9% omission rate), a decrease from 216 to 191 conditions (11.6%), and the loss of one of the 27 participants.

## Results

### Analysis

**Figure 5:**
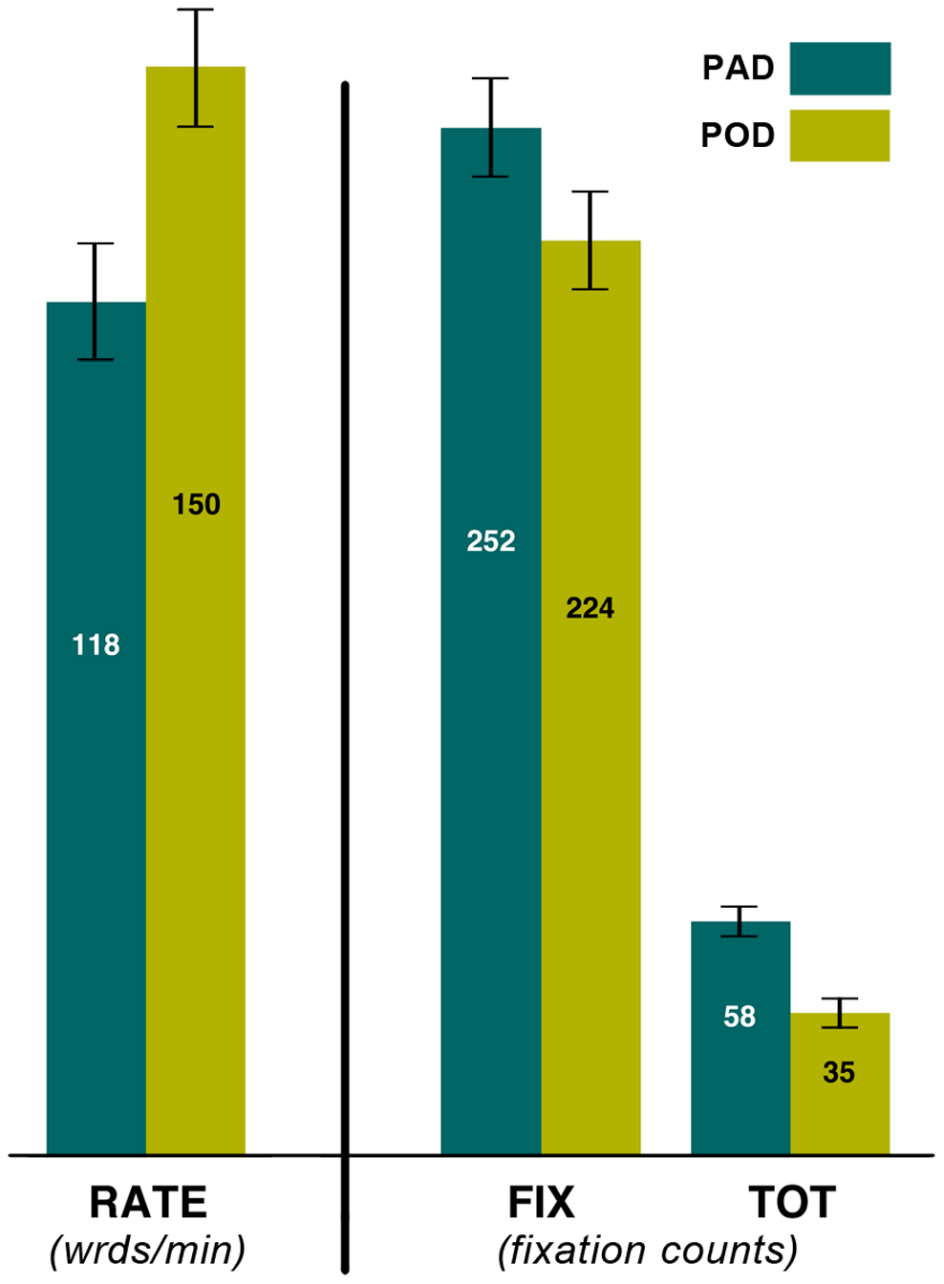
Short lines facilitate reading in dyslexia. When comparing the POD and PAD conditions, reading in the POD condition is faster (RATE), fixations are reduced (FIX), and the number of inefficient saccades substantially lower (TOT). These gains are observed at no cost to comprehension. (Error bars are +/−1 s.e.)

Our experimental design is of the ”repeated measures” type, in which all participants were measured in all conditions. This design was statistically modeled by a 2-level hierarchical linear model (HLM), with subject as the higher level. Our findings are summarized in Tables 2 and 3. Estimates of the means for significant main effects of Device are plotted in Fig. 5. The tables present only main effects models. However, two hypotheses involving interactions were also tested for each dependent variable: (a) that added spacing makes reading more difficult when linewidths are longer (“Device*Crowding” interaction), and (b) that highly compensated readers are less sensitive to effects of crowding (“Crowding*High Reading” interaction). None of the interaction models testing these hypotheses yielded a significant interaction. Hence they were omitted from the tables. (In Table 2, “better” indicates the direction advantageous for effective reading performance.)

**Table 2:** Results of hierarchical linear models: Primary oculomotor effects.

Dependent Variable	RATE		FIX		LEFT		TOT		UP		DN		OUT	
Effect	Coefficient	p-value	Coefficient	p-value	Coefficient	p-value	Coefficient	p-value	Coefficient	p-value	Coefficient	p-value	Coefficient	p-value
Device	31.8033	<.0001	−27.4919	<.0001	−26.1534	<.0001	−21.8221	<.0001	2.0392	0.0047	2.0219	<.0001	0.15	0.316
Crowding	−9.3233	0.0029	26.1471	<.0001	−0.03906	0.9794	−0.4034	0.8172	0.4225	0.5214	−0.9449	0.0144	0.1448	0.3328
Hand	1.4427	0.6135	6.8031	0.1992	1.9021	0.2213	1.8376	0.3037	0.2523	0.7041	−0.3073	0.4012	0.2208	0.1465
High Reading	32.3351	0.0581	−31.3949	0.2064	−4.0965	0.373	−3.1585	0.6638	0.559	0.8339	0.8534	0.3167	0.0187	0.9112

**Dependent Variables.** RATE: Reading speed in words/min (higher = better); FIX: Number of fixations (lower = better); LEFT: Number of regressive saccades (lower = better); TOT: Total number of inefficient saccades (lower = better); UP: Number of gaze motions up (lower = better); DN: Number of gaze motions down (lower = better); OUT: Number of gaze motions off-page (lower = better). **Effects.** Device (1 = POD, 0 = PAD); Crowding (1 = SPACED, 0 = NORMAL); Hand (1 = HAND, 0 = NO-HAND); High Reading (1 = yes, 0 = no).

**Table 3:** Estimated means of oculomotor variables.

Dependent Variable	Units	Estimate	Std. Err.	Estimate	Std. Err.
Device		*PAD*		*POD*	
RATE	wrds/min	117.79	8.0996	150.21	8.1231
FIX	number	251.87	12.0138	224.24	12.0697
LEFT	number	48.2701	2.6971	20.8142	2.718
TOT	number	57.5589	3.6281	35.0237	3.6486
UP	number	7.0379	1.2143	9.4516	1.2221
DN	number	2.0294	0.4196	4.2225	0.425
OUT	number	0.2268	0.1043	0.4193	0.107
**Crowding**		***NORMAL***		***SPACED***	
RATE	wrds/min	138.93	8.1044	129.08	8.1183
FIX	number	224.41	12.026	251.7	12.0576
LEFT	number	34.5929	2.7022	34.4914	2.713
TOT	number	46.5263	3.6327	46.0564	3.644
UP	number	8.0617	1.2161	8.4278	1.2203
DN	number	3.5727	0.4211	2.6791	0.4234
OUT	number	0.233	0.1054	0.4131	0.1059
**Hand**		***NO-HAND***		***HAND***	
RATE	wrds/min	132.65	8.1267	135.36	8.1004
FIX	number	236.93	12.078	239.17	12.0161
LEFT	number	34.3704	2.721	34.7139	2.6982
TOT	number	46.1598	3.6516	46.4229	3.629
UP	number	8.1284	1.2232	8.3611	1.2147
DN	number	3.2493	0.4255	3.0026	0.42
OUT	number	0.2492	0.1065	0.3969	0.1048

### Variations within Sample

To investigate potential effects of certain varying characteristics within our sample, we carried out supplementary analyses for four dependent variables: FIX, LEFT, TOT, and RATE. The additional independent variables were global report (VAS) (as described earlier), sight word efficiency (SW), and phoneme decoding efficiency (PD). Here, SW is the standard score derived from the number of sight words correctly read in 45 seconds from the TOWRE, and similarly PD is the standard score derived from the number of nonwords correctly decoded in 45 seconds, also from the TOWRE. These independent variables were entered in HLMs with Device, Hand, and Crowding. As before, observations flagged as faulty were deleted, leaving 25 participants in the analysis. Table 4 shows the significant main and interaction effects (with conditions) of these independent variables. Note that the shown significance (p = 0.0406) for the HAND main effect in the interaction model of TOT is merely a statistical artifact of the interaction.

**Table 4:** Summary of additional analyses: Influence of variation within the sample.

Dependent Variable	RATE		FIX		LEFT		TOT	
Effect	Coefficient	p-value	Coefficient	p-value	Coefficient	p-value	Coefficient	p-value
Device	−18.3532	0.3829	−**24.9336**	**<.0001**	−**26.3543**	**<.0001**	−**21.7398**	**<.0001**
Crowding	−**36.7951**	**0.0103**	**26.8898**	**<.0001**	−0.1273	0.9262	−0.1732	0.905
Hand	2.8915	0.3092	3.3127	0.4401	0.7349	0.5978	−15.2919	0.0406
PD	**2.008**	**0.0266**	−**3.2714**	**0.0072**	−**0.8358**	**0.0031**		
VAS	−4.6432	0.6601					0.8574	0.8805
VAS*Hand							**5.3656**	**0.018**
VAS*Crowding	**8.5659**	**0.0417**						
SW	**1.4986**	**0.0418**						
SW*Device	**0.6903**	**0.0086**						

**Device:** (1 = POD, 0 = PAD); **Crowding:** (1 = SPACED, 0 = NORMAL); **Hand:** (1 = HAND, 0 = NO HAND).

#### Hand

**Figure 6:**
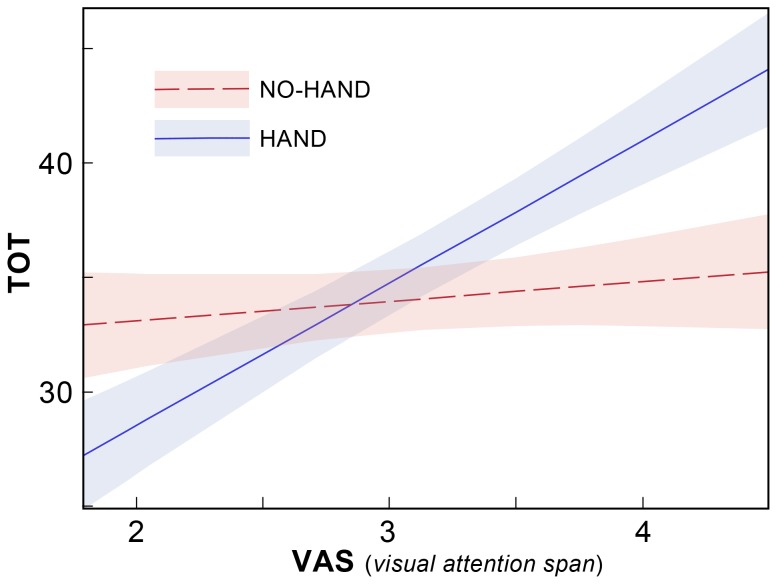
Holding the device in the hand alters erratic reading patterns. The observed interaction of Hand and global report (VAS) is plotted for TOT, the number of fixations that depart from efficient reading. A significant interaction of Hand*VAS was observed when TOT was taken as the dependent variable. Here, the TOT is shown as a function of VAS, the number of letters correctly identified on a six-letter global report paradigm. The NO-HAND condition is indicated in red, and HAND is indicated in blue. The figure shows that those with high scores on the global report task make more TOT errors when the device is held in the hand than when they do not hold it, while the reverse is true for those with low scores. When the hand is placed in the lap, variation in VAS makes little difference. (The colored shading indicates a confidence interval for this interaction, defined by a +/−1-sigma within-subjects standard error of the mean [72]. The graph is based on the POD and SPACED conditions.)

The hand condition (placing the hands near the device or not) clearly made no difference in any of the models with one important exception (see Table 4): we observe a significant interaction of Hand*VAS. Fig. 6 shows the shape of this interaction. For those with a high VAS score, TOT is higher in the HAND condition than in the NO-HAND condition. For those with low VAS scores, the opposite is the case.

#### Crowding

**Figure 7:**
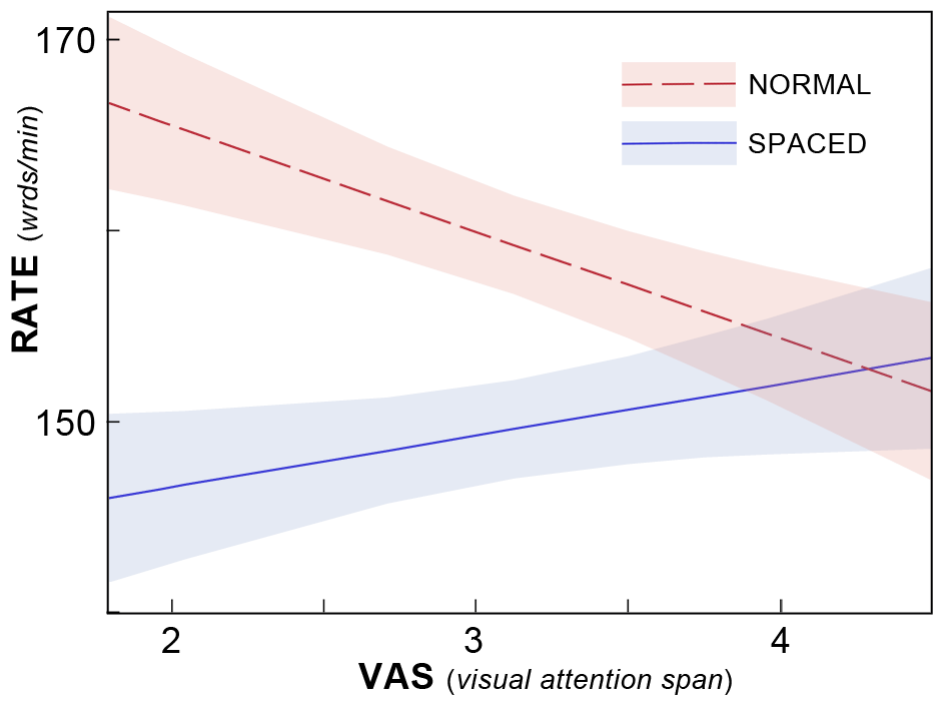
Extra letter spacing slows reading for those with low VAS. Interaction of Crowding and global report (VAS) is plotted for RATE, a measure of reading speed (see Fig. 4). A significant interaction of Crowding*VAS was observed when RATE was taken as the dependent variable. VAS is the number of letters correctly identified on a six-letter global report paradigm. Letter spacing makes little difference for those with high VAS scores. However, those with low VAS scores read faster using normal spacing. This suggests that increased letter spacing impedes oculomotor dynamics in readers likely to be characterized as most impaired. (Confidence intervals indicated as in Fig. 6). The graph is based on the POD and HAND conditions and median values of the continuous variables not part of the interaction.)

Significant main effects were observed for the letter-spacing conditions. Surprisingly, the normal spacing of text was favored over formats that reduce crowding in the majority of tracking variables where significance was observed (RATE and FIX). However, Table 4 shows a significant interaction of VAS*Crowding for RATE. The shape of this interaction is such that for those with high VAS scores, RATE is unaffected by the increased letter spacing (Fig.7). On the other hand, those with low VAS scores read faster under normal spacing, compared to the SPACED conditions.

**Figure 8:**
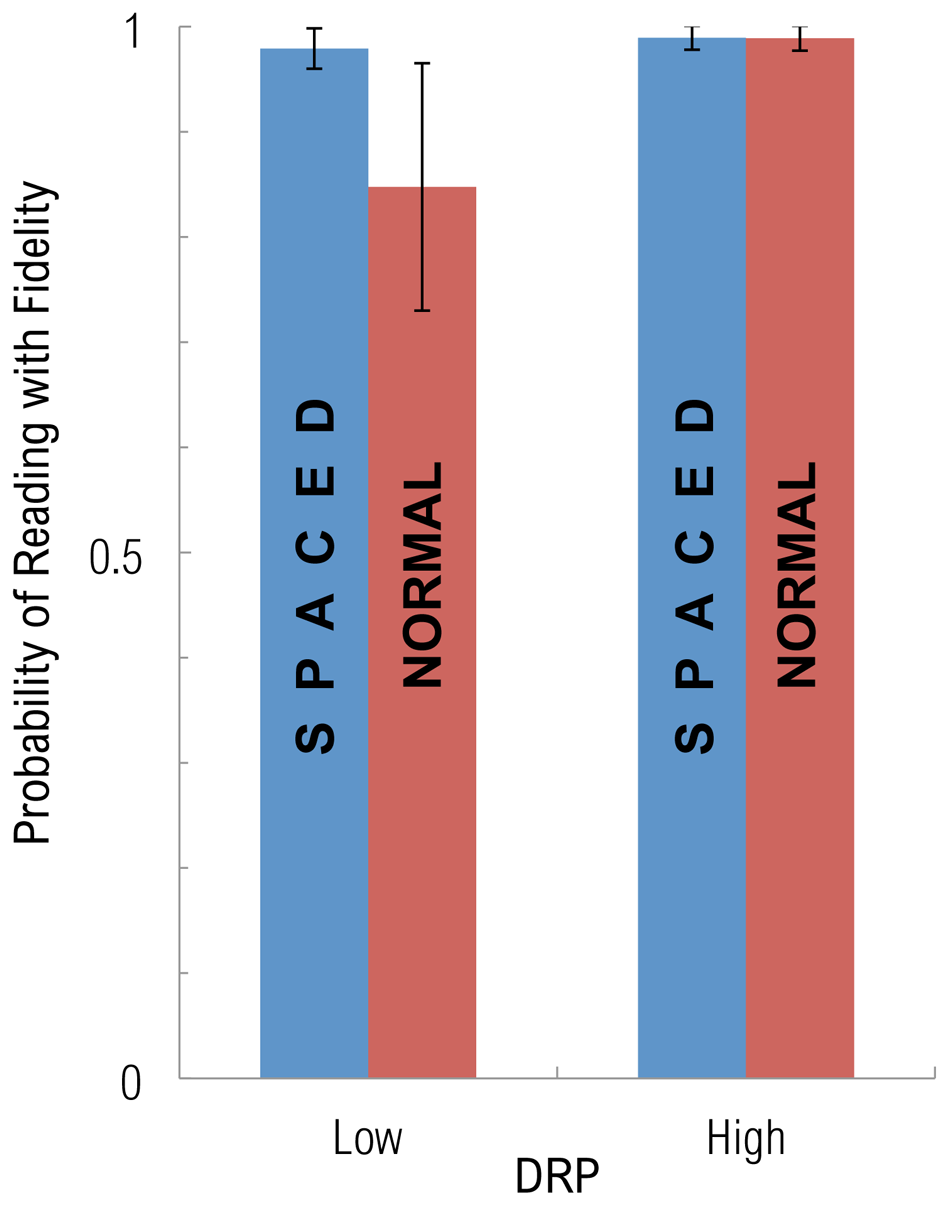
Extra letter spacing improves comprehension in those most impaired. The shape of the interaction of Crowding and reading levels for FIDELITY, a measure of reading comprehension, is plotted. A significant interaction of Crowding*High Reading was observed when FIDELITY was taken as the dependent variable. Degree of Reading Power (DRP) scores for each student, an a priori measure of their overall reading level, was used to create a dummy variable (High Reading) used to divide participants into high and low reading level groups. Those with high reading levels read well with either spaced or normal text, however, those most impaired read more effectively in the spaced condition. (Error bars indicate +/−1 s.e.)

Prior experiments [14] showed that when children with dyslexia (mean age 10.4 y) read aloud, they read more rapidly and with fewer errors when letter-spacing was increased. Our data did not show this expected benefit in RATE. And, while it is difficult to extrapolate findings observed in young children under conditions of reading aloud to results we report here, observed in high school students under conditions of silent reading, we considered the possibility that increased letter-spacing, rather than improving oculomotor dynamics, improves the accuracy of word decoding. To test this hypothesis, the dummy FIDELITY was entered as the dependent variable into hierarchical logistic regressions. Table 5 shows a main effects model (left) and a model with the “Crowding*High Reading” interaction (right). Here, “better” indicates the direction advantageous for effective reading performance. For ease of interpretation, the coefficients were exponentiated so that they can be understood as odds ratios (OR). (For example, the main effects model estimates the odds of achieving reading fidelity to be about three times as high for the SPACED condition as for the NORMAL condition.) We observed a marginally significant main effect of device (POD being better). Here, we suspect that the effect of device did not reach significance because most readers read at high levels of FIDELITY, and the underlying three-point scale used was too coarse to show a reliable effect. A significant effect of spacing was observed (SPACED being better), supporting the findings of [14]. In this instance, however, the interaction between spacing and reading level was significant. To understand the shape of the interaction, we converted the coefficients into least-square means that indicated the students’ probability of achieving fidelity (see Fig. 8). The good readers had no difficulty with either spaced or normal text: they almost always succeeded in either condition (98.8% SPACED, 99.0% NORMAL). For the weaker readers, by contrast, spacing did make a difference. Only in the spaced condition did they do almost as well as the good readers (97.7%); in the normal condition, they did worse (85.4%). This supports the hypothesis that the added spacing, used to reduce the effects of crowding, acts to facilitate word decoding–though this was apparent only among weak readers.

**Table 5:** Fidelity as dependent variable.

	*Main Effects Model*			*Model with Interaction*		
Dependent Variable	FIDELITY *(higher = better)*			FIDELITY *(higher = better)*		
Effect	Coefficient	OR	p-value	Coefficient	OR	p-value
Device (1 = POD, 0 = PAD)	*0.6603*	*1.94*	*0.0507*	0.6462	1.91	0.0624
Crowding (1 = SPACED, 0 = NORMAL)	**1.1074**	**3.03**	**<.0001**	0.04471	(1.05)	0.8761
Hand (1 = HAND, 0 = NO-HAND)	0.2285	1.26	0.2561	0.1989	1.22	0.3287
High Reading (1 = yes, 0 = no)	2.0516	7.78	0.1404	*2.774*	*(7.78)*	*0.0604*
Crowding(1)*High Reading(0)				**2.0841**	**(8.04)**	**<.0001**

#### Device

**Figure 9:**
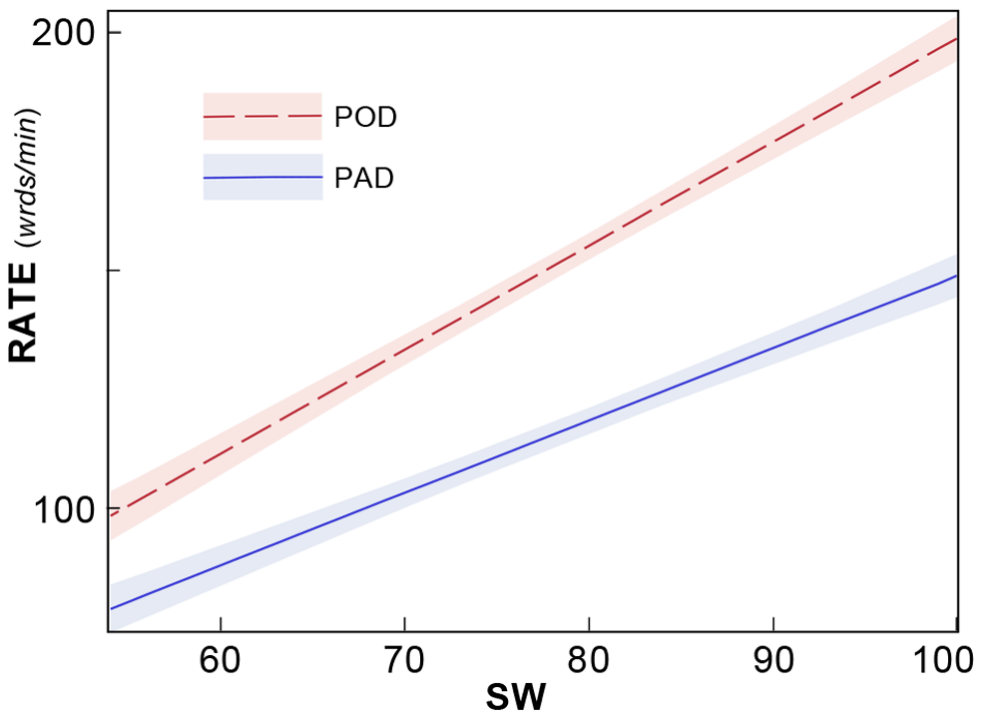
Speed benefit of POD is more pronounced in those with strong SW skills. Interaction of Device and Sight Word Efficiency (SW) is plotted for RATE. Participants read faster on the POD than on the PAD. As expected, those with high SW scores read faster than those with poor sight word skills. However, the speed advantage of the POD is more pronounced in those with high SW scores. (Confidence intervals indicated as in Fig. 6.)

Considering variables measured by eye tracking, indicative of the mechanical efficiency of reading, we found a number of significant effects. Significant main effects favoring the POD condition were observed in most eye tracking variables, including speed (RATE), fixation count (FIX), and tracking errors (TOT), and these are shown in Fig. 5. With the added variables, a significant interaction of SW*Device is observed (see Table 4 and Fig. 9), indicating that the speed advantage of POD over PAD is more pronounced for those with high SW than for those with low SW.

Not surprisingly, significant main effects of PD are also observed (see Table 4). Those with high PD scores made fewer fixations, exhibited fewer horizontal regressions, and read with greater speed.

To summarize, the most salient of our findings provide strong support for the hypothesis that the shortened lines used in POD lead to more efficient oculomotor dynamics, compared with the longer linewidths used in PAD.

## Discussion

### Hand: Holding e-readers in the Hand Regulates Reading in those Most Impaired

Research shows that the hand alters the allocation of attention during reading in ways that are complex, to benefit certain functions, but hinder others [26]–[28]. Expected benefits of holding an e-reader are to focus attention to detail [10], minimize distractions [9], and thereby presumably improve single word decoding and perhaps comprehension. On the other hand, given that shifts in attention are slowed when objects are examined in the hand [9], it is also possible that holding the e-reader would slow attention shifts made during reading and introduce a cost in reading speed (RATE) in our experiment. Though none of these effects were observed in our experiment, we did find that use of the hand interacted significantly with VAS in predicting TOT, the total number of irregular gaze movements (unexpected saccades made during reading in directions up, down, back, or off the page). Those with poor VAS scores were better able to regulate their oculomotor dynamics when the device was held in the hand, while the opposite was true of those who had strong VAS scores (see Fig. 6).

Low VAS in dyslexia has been characterized as a visual attention span deficit [29], [30], and is thought to reflect a diminished sensitivity to the number of characters perceived at a glance during reading. The global report task briefly flashes a string of widely-spaced letters at fixation and scores the number identified, tapping into abilities to distribute attention over a span of about 4°. However, by requiring participants to recall the letter string, the task also invokes processes for working memory [31], and letter naming [32], functionalities sometimes impaired in dyslexia. And because no backward mask was used in our implementation, iconic visual memory also plays a role in our measure of VAS. (Given that the global report task presents letters that are widely spaced, crowding plays less of a role in this task.) Our observations suggest that when e-readers are held in the hand. this regulates oculomotor dynamics in those with dyslexia whose abilities for distributed attention (or perhaps other factors implicated in this task) are impaired. The hand may shield attention from visual interference [9] to improve distributed attention used to guide oculomotor tracking in those with low VAS. However, it is not immediately obvious why those with high VAS might perform more poorly when the device is held, making this an interesting question for future research.

### Crowding: Increased Letter-spacing can Improve Comprehension, but at a Cost

When we manipulated crowding by increasing the spacing between characters, we reliably observed expected advantages of letter spacing [14] for comprehension, but primarily in the readers with dyslexia who were most impaired. While the stronger readers had no difficulty with either spaced or normal text, for the weaker readers, by contrast, spacing did make a difference. When the text was spaced, comprehension among weaker readers was indistinguishable from that of the stronger readers, but when the spacing was normal, the weaker readers performed worse.

In contrast, increased letter spacing failed to produce similar benefits on the oculomotor variables. Observed main effects (Table 2) indicate that students made more fixations (FIX) in the SPACED than in the NORMAL condition, and the speed advantages reported by [14] were not observed in our data. If anything, students read more slowly when text was spaced (Fig. 7). While spacing made little difference to those with high VAS scores, those with low VAS scores read faster when text was normally spaced. The only benefit of letter spacing we observe is that the number of downward errors (inappropriate glances down to text below the line currently read) is decreased. However, given only about one percent of all fixations are downward errors (see Table 2), this effect is of negligible importance in our study.

We speculate that the reason our study only partially reproduced findings of [14] is that the nature of the format manipulation differed in the two studies. In the prior experiments of [14] letter spacing and line spacing were allowed to co-vary, while in the present study, line spacing was held fixed, and only letter spacing was altered in the Crowding condition. Furthermore, the prior study held margins fixed as letter spacing was manipulated. This had the effect of altering linewidth (using our definition of words per line), and introducing an important confound with effects we find modulate oculomotor dynamics. Thus, it is difficult to know from the prior study the degree to which the advantages it reports are due to letter spacing, as opposed to other effects. We infer from this that, while increased letter spacing likely reduced reading errors in the previous study by reducing inter-letter crowding, the improvements in speed they report came primarily from the benefits of added line spacing and decreased linewidth. Based findings we report here, we conclude that while letter spacing may facilitate decoding of individual words (and thus improve comprehension), it does not in itself improve the efficiency of oculomotor dynamics in reading.

### Device: Shorter Lines Reduce the Incidence of Regressive Saccades

Overall, we find that reading using POD is significantly more effective than using PAD in virtually all measures of reading performance used. For example, comparing the variables FIX and LEFT, as many as one in five saccades made during reading in the PAD condition are regressive horizontal saccades (LEFT), directed backwards in the line. However, in the POD condition, the number of those inefficient saccades is cut in half. Furthermore, almost every other significant measure of reading is improved. Notably, the instantaneous reading rate (RATE) is faster by 27% in the POD condition compared with PAD (see Fig. 5). These improvements in oculomotor dynamics do not occur at the expense of comprehension. If anything, marginally significant advantages for reading fidelity are also associated with the POD condition. The only exception, where PAD holds an advantage, is that downward directed errors (DN) increase in the POD condition. Though significant, given that these vertical events represent only 6% of all tracking errors, they have only a marginal influence on reading performance. When all tracking errors are taken into account (TOT), the POD condition produces 40% fewer such errors compared with the PAD. Therefore, the POD is strongly advantageous.

The strong results favoring the POD condition are most readily understood as a consequence of differences in the average number of words displayed in a line (linewidth). In the POD the linewidth is 2.19 words per line (wpl), displaying an average of 12.7 characters per line, while in the PAD condition the linewidth is five-fold larger, at 11.6 wpl (67.2 characters per line). Previously, eye tracking was used to investigate the effects of linewidth in reading [33], [34] and it was shown that, when linewidths of 18 wpl are compared with those of 9 wpl, shorter lines tended to decrease reading time, increase retention, and produce fewer regressive saccades (eye movements backwards along a line). However, an earlier eye tracking study [33] found that the benefit of shorter lines breaks down when linewidths fall below an optimal intermediate length. When long (23 wpl), intermediate (10 wpl), and short (4 wpl) linewidths were compared, it was found that reading efficiency optimal for intermediate lengths. In contrast, in our sample reading improved when lines were shortened from 12 wpl (PAD) to 2.1 wpl (POD). Given that the linewidths used in the POD condition, advantageous here, fell far below the optimal length observed in prior studies, we suggest that the observed discrepancy is attributable to dyslexia in our sample.

To the best of our knowledge, linewidth has not been previously studied in dyslexia. However, a well-known word length effect [35] is observed in transparent orthographies, where vocal reaction times to stimulus onset increase monotonically with greater word length in dyslexia. This has been attributed to eccentricity dependent deficits in crowding that would impair recognition of longer words [36]. Given that linewidths used in the POD condition approach single word reading (1 wpl), the advantages we observe favoring shorter lines may be similarly related to an abnormal response to crowding in dyslexia. This hypothesis is discussed in more detail in the sections to follow.

Linewidths alter regression within lines, not between. It is a puzzle why patterns of regression sometimes appear to be highly regular, as if almost automatic, in some of our students with dyslexia, and why such regressions are effectively controlled by formatting manipulation (see Fig. 2). In dyslexia, the incidence of regressive saccades can be higher by a factor of two [37]. Because we observe that the incidence of regression is strongly controlled by manipulating linewidth, we consider this effect in greater detail.

At first blush, it may not seem surprising that the narrow formats control horizontal regressions, because it is obvious that, as formats are narrowed, the probability that a previously fixated word will occur on any given line will decrease, providing fewer targets for regression on the line. In other words, as the lines become short, the chance of regressing horizontally to a previously encountered word on a line decreases. For the PAD condition the probability a word can be found immediately to the left is 91%, but it is only 54% for the POD. And referring to Table 3, the incidence of horizontal regression is (LEFT/FIX) is 19% for PAD, and 9% for POD, a ratio roughly proportional to the probability determined by linewidth. Thus, indeed, the incidence of horizontal regression appears to roughly scale with the linewidth, as expected.

**Figure 10:**
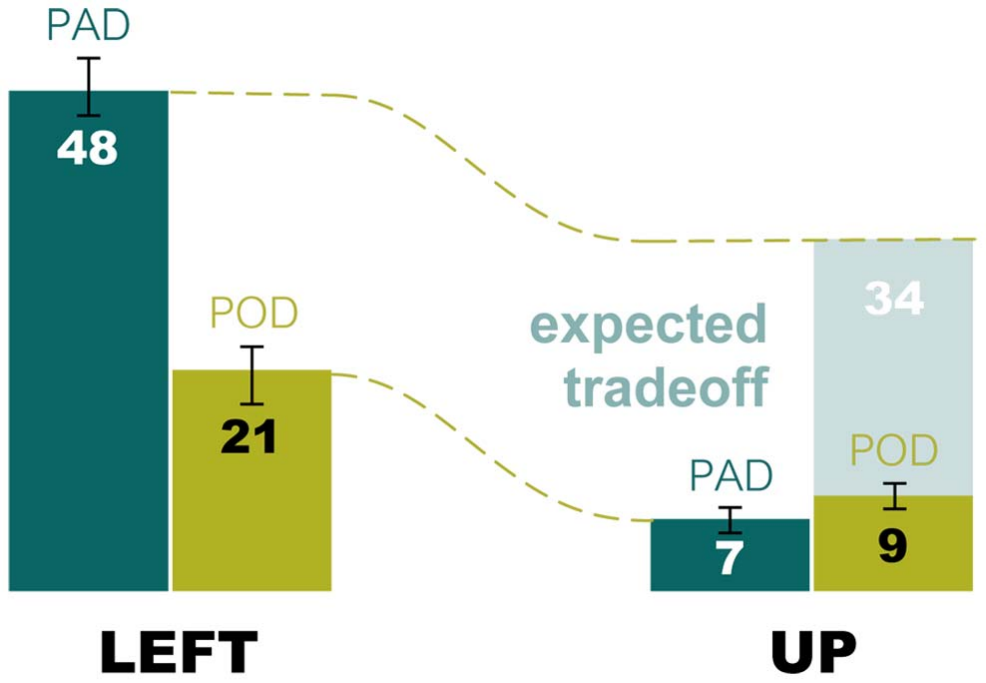
Lapses in high level lexical processing rarely cause regression. There are twice as many horizontal regressions (LEFT) in PAD as in POD. If the mechanism for regression was entirely due to lapses in conceptual understanding, we would expect regressions made horizontally to trade off against regressions made upwards (UP), as margins are made narrow in the POD condition. And yet, this expected tradeoff is not observed. Narrowed columns halve the number of horizontal regressions, but hardly increase the number of regressions upward. This indicates that the mechanism for regression is restricted to the line, and is likely proximal to fixation. (Error bars are +/−1 s.e.)

However, another consequence of breaking the text into short lines is that, when formats are narrowed, opportunities for re-inspection across line boundaries increase. Readers normally issue regressive saccades to clarify meaning, or otherwise address lapses in understanding, and we would expect that formatting manipulations would not alter the incidence of regressive saccades, driven by such demands of lexical analysis. Therefore, an increased number of vertical regressions in POD is expected to be the price to be paid for the decreased number of horizontal regressions in that condition. Yet this is not observed (see Fig. 10). Though the incidence of horizontal regressions drops by a factor of two, from 19% for PAD to 9% for POD, the incidence of upward saccades in POD does not scale up to make up this difference. Although the incidence of upward-directed saccades does indeed increase from 3% for PAD to 4% for POD, this difference is an order of magnitude short of what would be expected if the regressions were driven primarily by lexical analysis and thus would bridge across lines. Therefore, while the decrease, from PAD to POD, in the incidence of leftward saccades can be almost entirely explained by the change in linewidth, the lack of an associated increase in vertical saccades cannot be explained unless the phenomenon driving the regression is something local to the fixated word that acts horizontally within a line. Our data suggest, furthermore, that even if local, the dominant mechanism for regression is less likely a consequence of decoding issues at the site of the fixated word. We believe this to be the case because, when reading in the POD condition, regression drops by a factor of two, at no cost to comprehension (comprehension is observed to marginally improve). Given that readers often regress to correct for lapses in understanding, and thus clarify meaning, we would expect comprehension to suffer if a text manipulation caused regression rates to drop. The fact that this does not occur further suggests that shortened linewidths act to limit confusion at the sight of the fixated word. Based on the forgoing, we suggest that a possible explanation, consistent with the observations in this study, is that short lines act to improve comprehension at the fixation site by reducing the likelihood that a previously fixated word can be found immediately adjacent to the fixation site. We explore this suggestion further, in the context of the literature, in the section below.

### Do Deficits in Attention and Crowding Collude to Drive Regression?

If, as suggested above, the seemingly automatic generation of horizontal regression we observe (see Fig. 2) is not caused by higher-order lapses in sense-making, what then could drive such patterns of regression that can be controlled by the use of short lines? Given that these regressions are largely horizontal and only rarely breach lines, an oculomotor explanation tied to the direction of reading must be considered. While our data do not speak directly to this question, in this section we turn to the literature to proffer a speculative account meant to generate plausible hypotheses that can motivate future research.

#### (a) Good readers maintain attention on the uncrowded span as the gaze shifts in reading

Reading speed is fundamentally limited by the visual span, defined as the number of characters able to be accurately perceived at a glance [38]. It has been shown that the visual span is in turn determined by crowding, and thus equivalent to the number of characters in the uncrowded span [39]. Furthermore, crowding is isotropic about fixation, once the moderating effects of attention are taken into account [40], [41], so the uncrowded span is expected to be yoked to fixation as the gaze advances during reading.

**Figure 11:**
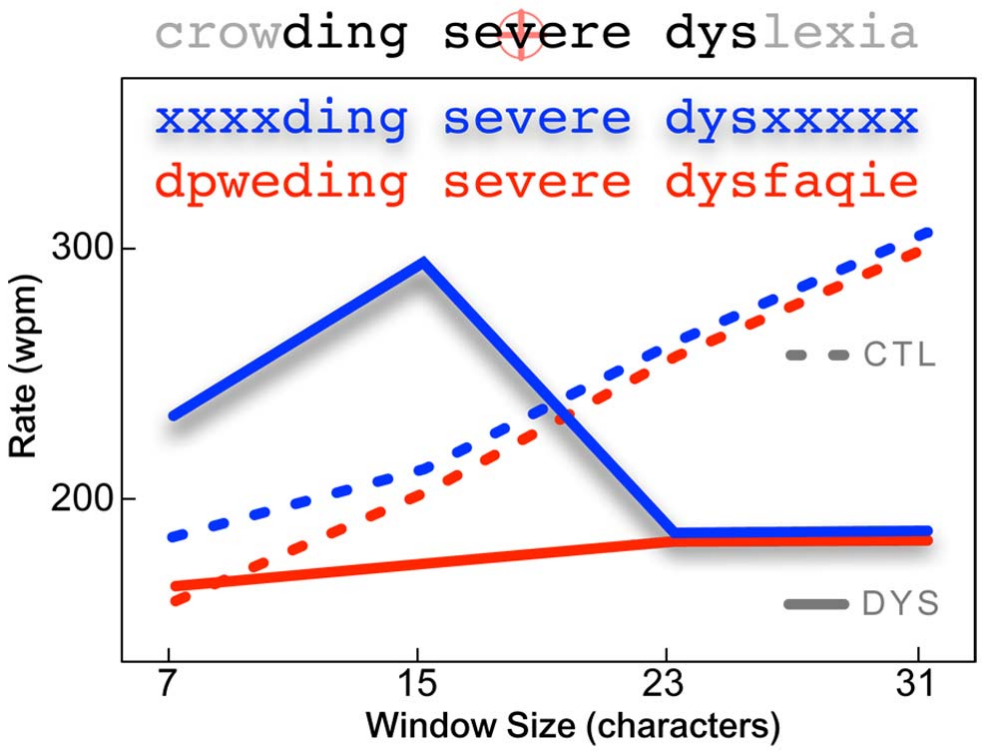
A case study of “selective attentional dyslexia” from Rayner, et al., 1989. A gaze-contingent display was used to mask letters on either side of fixation. When the mask was composed of X’s (blue) the person with dyslexia (solid line) outperformed typical reading controls (dashed line), reading as if unimpaired when the window size was 15 characters. But, when the window was formed by randomly replacing letters (red), the dyslexic individual performed poorly. We interpret this to suggest that the individual with dyslexia was unable to maintain attention on the “uncrowded span” [39] as the gaze advanced during reading, unless it was clearly demarcated using X’s in a gaze contingent display. When this was done, the person could read at near normal rates. (Data from [42].)

Presumably strong readers are able to maintain attention to the uncrowded span as the gaze shifts during reading. Whether or not this is the case in dyslexia is not known. One of the few extant examples of research that speak to this question comes from a case study of an individual with “selective attentional dyslexia” [42]. In this study, a gaze-contingent display was used to replace letters with X’s outside a span of characters centered on fixation during reading (see Fig. 11). The individual with dyslexia in this case study was ordinarily a very slow reader. Remarkably, when reading using a window of 15 characters centered on fixation, this person was able to read at normal rates. However, when random characters were used to mask letters outside this window, the person with dyslexia read very poorly compared to the controls. We interpret these observations to suggest that this individual ordinarily read poorly because he was unable to maintain attention to the uncrowded span as the gaze advanced. Only when the uncrowded span was artificially delineated using X’s was the person able to read at normal speeds. When random characters demarcated the uncrowded span, the person was unable to reject these irrelevant peripheral letters and attend to the uncrowded span centered at fixation.

#### (b) Good readers reject previously read text located to the left of fixation

Using gaze-contingent paradigms it has been shown that, in typical readers, the available perceptual span used during reading is asymmetric, extending a few letters to the left of fixation, but as many as 15 letters toward the right [43]–[45]. Even though crowding typically makes it difficult to discern text further than about seven letters from fixation [39], text perceived in the parafovea, to the right of fixation, provides word shape and other information that provides a preview benefit used in reading [46]. On the other hand, text located to the left of fixation, in fields previously fixated, serves no practical purpose in normal reading [43]. Despite this, there is evidence that typical readers nevertheless attend to the text located to the left [47]–[49]. Evidence for this comes from gaze contingent boundary change paradigms used to alter words left of the fixation target in mid-saccade. In one such experiment [48], researchers found that in cases where readers skipped the altered word, they regressed to the skipped word more often when this word was manipulated so as to cause a conflict with expected meaning, demonstrating that attention is allocated left of fixation in normal reading. Other experiments [49] similarly manipulated words to the right and left of the fixation target and found that the processing of orthographic information to the right and left of a fixated target is functionally independent. Furthermore, in cases where regressive saccades are issued, the perceptual span used in reading is observed to change, so as to include information further to the left, immediately prior to the onset of a regressive saccade [50]. Thus, though attention directed to the right of fixation ordinarily serves to guide eye movements and otherwise provide a preview benefit in reading, people also independently attend to the text left of fixation. Moreover, though attention is spatially biased in the forward direction [43]–[45], to reject perception of text previously read, people are able to spread their attention further to the left in cases where regression is warranted.

#### (c) The allocation of attention to the left and right differs in dyslexia

**Figure 12:**
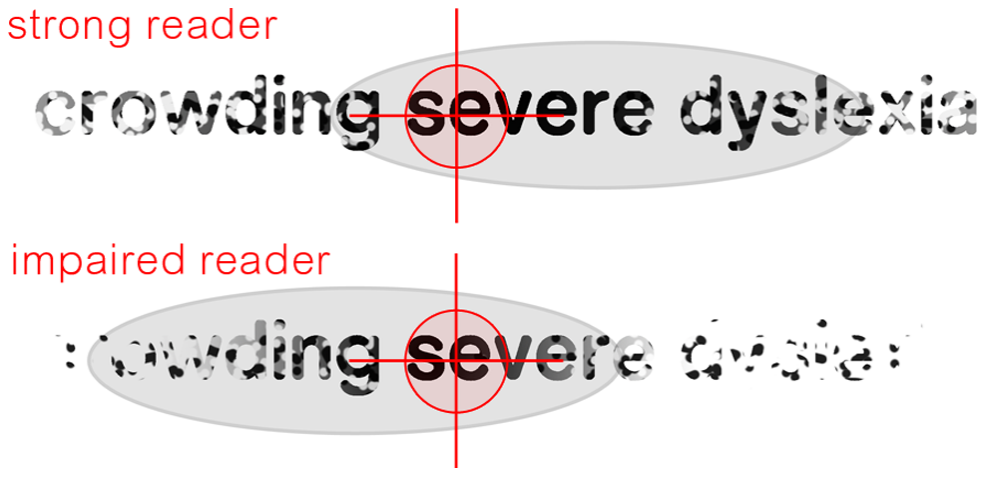
Schematic illustration of effects of attention and crowding in effective and ineffective readers. In typical readers, attention (grey oval) is primarily directed to the direction of reading, to provide a parafoveal preview benefit, and reduce attention to words previously inspected. In dyslexia, we propose that sluggish attention shifting causes attention to be slow to disengage from previously fixated locations, effectively spreading attention to the left of the fixated word. Given that crowding (indicated with stippling) is more severe in dyslexia, text perceived to the left is likely to be misperceived, increasing the potential for confusion.

There is considerable evidence suggesting that attention is lateralized differently in dyslexia (e.g., [51]–[59]). Therefore, it is natural to assume that the balance of attention allocated to the left and right of fixation during normal reading will be biased differently, as well. For example, when words are flashed on either side of fixation, while typical readers show a well-studied bias for word recognition favoring the right visual field, this bias has been observed to reverse in dyslexia [56], implying greater sensitivity to text located to the left. Furthermore, given that attention shifting is sluggish in dyslexia [13], [60]–[63], such a bias will be exaggerated by the dynamics of reading. In this case, as the gaze advances from one word to the next, attention will be slow to disengage from previously fixated words, left of fixation, enhancing their perception. (see Fig. 12). Thus, normal attentional processes that allow perception of words to the left of fixation during reading [47]–[49] are expected to be exaggerated by the dynamics of reading in people who exhibit sluggish attention.

#### (d) Previously fixated words become crowded when the gaze shifts during reading

Much of the research on crowding has been done at fixation, and less is known about the influence of eye movements on this process. When eye movements are considered, it has been shown that in preparation for a saccade, object discrimination in peripheral vision is effectively enhanced at the crowded target, beginning as early as about 50 ms before a saccade is issued in the direction of this object [64]. Therefore, we can expect that as the gaze advances from one word to the next during normal reading, crowding at the site of the target word is rapidly diminished in advance of the saccade. However, once the previously fixated word falls to the periphery, given that crowding increases with eccentricity [65], the previously fixated word will become crowded. Crowding will therefore abruptly alter the orthographic percept of the previously fixated word so as to make this word difficult to discern. To the extent that typical readers attend to words to the left during normal reading, such words will necessarily be affected by peripheral crowding. Therefore, if crowding is more severe in dyslexia, as is reported [14]–[16], [36], [66], and/or sensitivity to previously fixated sites is greater in dyslexia (as we speculate above), we would expect such effects of crowding to be exaggerated in dyslexia.

#### (e) Does perception of crowding on the left induce regression?

**Figure 13:**
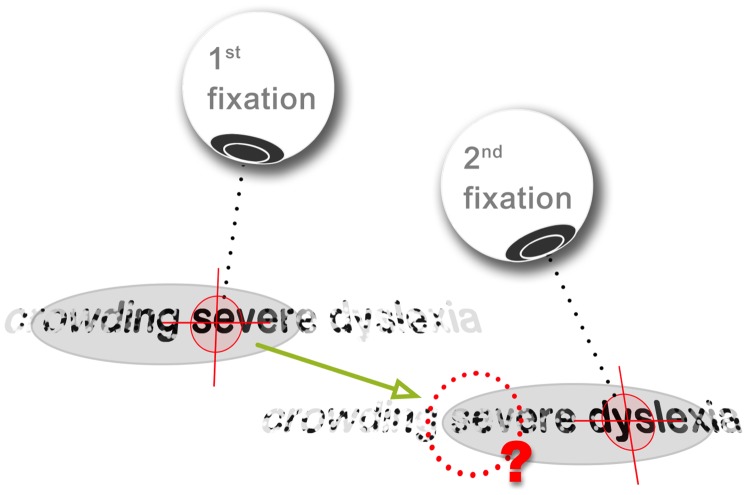
Proposed mechanism driving regression. During reading a short gaze shift is made from the word “severe” in the first fixation, to “dyslexia” in the second. We suggest that due to attention deficits, the word “severe,” left of fixation, is perceived even after the fixation has advanced. However, because this word is now in the periphery, crowding alters its percept. If this sudden change in orthographic percept triggers a cognitive dissonance that calls the word’s previous interpretation into doubt, this can encourage a regressive saccade to re-inspect the word. We refer to this process as a “crowding induced regression (CIR).”

If a change in percept due to crowding in a previously fixated word causes readers to doubt their prior interpretation, a regressive saccade may be issued to clarify meaning (see Fig. 13), in a process we term “crowding induced regression” (CIR). We suggest that typical readers, who have a strong command of attention, are able to inhibit CIR by casting their perceptual span forward during reading [43]. However, for those with sluggish attention deficits, crowded words to the left of fixation are more strongly attended, increasing the incidence of CIR. This issue is further compounded in those for whom crowding is severe [14]–[16], [36]. We speculate that this mechanism is responsible for the seemingly automatic patterns of regression seen in some people with dyslexia, where regressions occur approximately once every three fixations (see Fig. 2).

**Figure 14:**
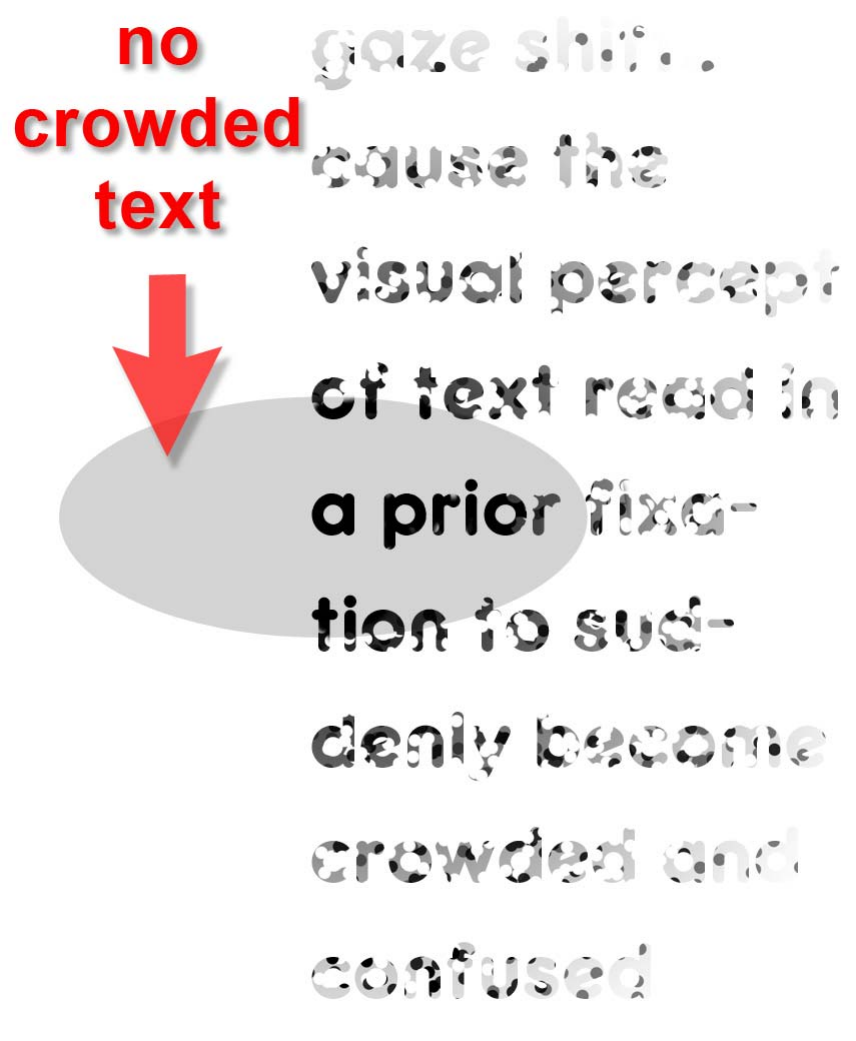
Narrow lines reduce the chance crowded text is perceived left of fixation. The grey oval illustrates a “window” of attention spread that includes sensitivity to the left of fixation. Stippling is used to suggest the effects of crowding. The probability that crowded text can be attended to the left of fixation –in fields attended in the previous fixation– is reduced when text is formatted as narrow columns.

In this framework, short lines reduce the number of regressions, and generally improve reading speed and comprehension, simply by reducing the probability that crowded text in locations previously fixated can be perceived (see Fig. 14). Given that CIR regressions are issued to clarify meaning, the extent to which diminished linewidth will enhance reading will vary depending on the reader’s familiarity with the text. Though aspects of this interpretation are undoubtedly speculative, this raises a number of interesting hypotheses, and these need to be investigated through future research (see Conclusions).

### Other Considerations

#### Inhibition of return

Could the findings be alternatively explained by deficits for inhibition of return (IOR) in dyslexia? IOR is a phenomenon in which saccades generated to targets at previously attended locations are delayed [67]. When measured at fixation, using covert exogenous orienting paradigms, IOR response has been shown to be impaired in dyslexia [53]. A prediction of this finding is that while those exhibiting a robust IOR response would be slow to re-inspect previously fixated text, those with poor IOR response would not show this inhibition, and thus be more likely to rapidly issue a regressive saccade toward words immediately to the left of the current fixation. This suggestion is supported by evidence showing that those with weakly developed IOR generate shorter regressive saccades than those who exhibit a strong IOR response [68]. Thus, the IOR account can successfully explain why short regressions (such as those seen in Fig. 2) are suppressed in typical readers, but not in those with dyslexia. However, while the IOR explanation is consistent with the observations here, it is difficult to understand how this mechanism alone can account for them. In particular, while IOR explains why short regressions are not inhibited in dyslexia, it says nothing about what may cause the regression in the first place. As discussed above, given that horizontal and vertical regressions are not observed to trade off as linewidths are narrowed, the cause of regression –controlled by the short linewidths– cannot be the result of cognitive lapses (e.g., comprehension problems or memory lapses). To explain this, a mechanism other than IOR (such as CIR) is needed to generate the regressions.

#### Effects of letter spacing

If extra-large letter spacing reduces crowding, would this also reduce the incidence of CIR events? We would not expect this to be the case. Crowding increases with eccentricity [15]. Therefore, a given letter spacing chosen to just relieve crowding within the angular span of a typical word when it is viewed at fixation, will be insufficient to counteract crowding when this word is perceived in the periphery. Therefore, letter spacing does little to prevent CIR events. Indeed, this is what is observed (Table 5). If anything, spacing introduces oculomotor costs: it significantly slows reading by increasing the number of fixations (Table 2; RATE and FIX).

### Conclusions

A number of questions remain unresolved, suggesting further research. An interesting question is whether the advantages of short lines found here in a sample of students with dyslexia, carry over to typical readers. Given that we observe that within-subjects characteristics modulate the observed effects, we would expect such would be the case. This question is especially important in view of our proposal that sluggish attention shifting enhances attention to the left as the gaze advances. Experiments are needed to investigate this in greater detail, using gaze contingent paradigms similar to those of [42], characterizing those with and without attention deficits. In addition, care is needed to resolve confounds of letter spacing, line spacing, and linewidth in relation to the observed effects. Because we expect incidence of regression to scale in proportion to the number of words per line, it would be informative to test this effect at a variety of linewidths. Lastly, the allocation of attention, as well as the effects of crowding, traditionally investigated at fixation, needs to be reconsidered under conditions that better simulate the dynamics of reading. A strong test of our hypotheses would use a paradigm analogous to [69] to investigate attention and crowding in dyslexia under conditions of gaze motion.

While reformatting the page significantly improves reading in those with dyslexia, we emphasize that this alone cannot address all of the factors known to impede reading in this disorder. Altering spatial formatting can only partially alleviate factors affecting the temporal dynamics in reading such as slowness caused by sluggish attention shifting [60], difficulties accessing phonological representations of words [70], or latencies in naming [32], each of which can act, independently of the effects addressed here, to impair reading. Furthermore, given that people’s reading characteristics vary [71], it is reasonable to expect that the benefits of reformatting the page, will likely vary with individuals, as is observed here.

In conclusion, we note that in the century since dyslexia was first described, methods used for reading have undergone very little revision. However, with the widespread adoption of e-readers and other digital technologies, reading methods are rapidly evolving, and this offers an opportunity to reverse historically imposed constraints on reading, whose impetus was driven largely by technological limitations that are no longer relevant. As this study demonstrates, even relatively minor changes in the formatting and display of text, when done incisively, can lead to significant improvements in reading among those who otherwise struggle. The interactions found in this study further suggest that different readers may benefit from different reading formats and modalities. Here, the flexibility afforded by e-readers provides a critical advantage: Electronic text can easily adapt to the needs of individuals. Therefore, by reinventing reading in this digital age, everyone may be able to gain, and impairments in reading may cease to be a barrier for many people with dyslexia.
